# Peptide microarray of pediatric acute myeloid leukemia is related to relapse and reveals involvement of DNA damage response and repair

**DOI:** 10.18632/oncotarget.27086

**Published:** 2019-07-23

**Authors:** Hasan Mahmud, Arja ter Elst, Frank J.G. Scherpen, Tiny Meeuwsen-de Boer, Kim R. Kampen, Valérie de Haas, Victor Guryev, Maikel M. Peppelenbosch, Steven M. Kornblau, Eveline S.J.M. de Bont

**Affiliations:** ^1^ Stephenson Cancer Center, The University of Oklahoma Health Sciences Center, Oklahoma City, OK, USA; ^2^ Department of Pediatric Oncology/Hematology, Beatrix Children’s Hospital, University Medical Center Groningen, University of Groningen, Groningen, The Netherlands; ^3^ Dutch Childhood Oncology Group, Stichting Kinderoncologie Nederland, The Hague, The Netherlands; ^4^ Laboratory of Genome Structure and Aging, European Research Institute for the Biology of Aging, University Medical Center Groningen, University of Groningen, Groningen, The Netherlands; ^5^ Department of Gastroenterology and Hepatology, Erasmus Medical Center, Rotterdam, The Netherlands; ^6^ Department of Stem Cell Transplantation and Cellular Therapy, University of Texas, MD Anderson Cancer Center, Houston, TX, United States

**Keywords:** AML, leukemia, peptide microarray, DNA damage and repair, signal transduction

## Abstract

The majority of acute myeloid leukemia (AML) patients suffer from relapse and the exact etiology of AML remains unclear. The aim of this study was to gain comprehensive insights into the activity of signaling pathways in AML. In this study, using a high-throughput PepChip™ Kinomics microarray system, pediatric AML samples were analyzed to gain insights of active signal transduction pathway. Unsupervised hierarchical cluster analysis separated the AML blast profiles into two clusters. These two clusters were independent of patient characteristics, whereas the cumulative incidence of relapse (CIR) was significantly higher in the patients belonging to cluster-2. In addition, cluster-2 samples showed to be significantly less sensitive to various chemotherapeutic drugs. The activated peptides in cluster-1 and cluster-2 reflected the activity of cell cycle regulation, cell proliferation, cell differentiation, apoptosis, PI3K/AKT, MAPK, metabolism regulation, transcription factors and GPCRs signaling pathways. The difference between two clusters might be explained by the higher cell cycle arrest response in cluster-1 patients and higher DNA repair mechanism in cluster-2 patients. In conclusion, our study identifies different signaling profiles in pediatric AML in relation with CIR involving DNA damage response and repair.

## INTRODUCTION

Acute myeloid leukemia (AML) forms a spectrum of diseases that share clinical and pathological features. Improvements in chemotherapeutic regimens and supportive care have increased overall survival rates in younger patients (<15 years of age) to more than 60%, however, 40% ultimately relapse requiring salvage therapy [1, 2]. Extensive research, including genomic sequencing in the TCGA project, has revealed many previously unknown recurrent mutations and additional mechanistic insights such as alternations in the epigenetic status during leukemogenesis. Based on these new findings classification has evolved from the French-American-British system (FAB), which was driven by morphology and later surface markers, to the current World Health Organization (WHO) classification which incorporates the current well-defined karyotypical, mutations and genetic abnormalities. The WHO system, while prognostic in some groups, does not guide therapeutic recommendations [1, 3]. AML leukemogenesis and the regulation of leukemic cell proliferation and survival is the net result of all genetic, epigenetic and environmental factors present in the leukemic blast manifesting through canonical or non-canonical utilization of the existing signal transduction pathways in those cells. Deregulated protein phosphorylation by aberrant protein kinase activity is frequently observed in cancer, including leukemia [4–10]. Aberrant activity of multiple signal transduction pathways has been associated with a worse prognosis [11]. Unlike chronic myeloid leukemia (CML), where single treatment with BCR-ABL inhibitors has been proven very successful, the use of single kinase inhibitors in AML has not dramatically altered outcomes as in CML [12–14]. The failure of single kinase inhibitor therapy is likely due to defensive cellular adaptations that utilize alternative metabolic routes, or redundancy in pathways that provide escape mechanism [15–16]_._ Therefore, improved therapy of AML will require therapeutic agents directed against the perturbed pathways downstream of these events.

To address this, we recognized the need to develop a global map of the activation patterns going on in all of the signal transduction pathways within a cell. Therefore, we utilized a high-throughput peptide microarray array and investigated the peptide activation of 976 peptides simultaneously and quickly to generate a global map of pathway activation in pediatric AML. Unsupervised clustering clearly divided the AML samples into two different profiles, named cluster-1 and cluster-2 of which cluster-2 was strongly related to a higher cumulative incidence of relapse (CIR). In addition, cluster-2 samples showed to be significantly less sensitive to various chemotherapeutic drugs than cluster-1 samples. The identified activated peptide patterns of two clusters were independent of patient characteristics such as FAB, WHO classification with recurrent genetic abnormalities and mutations. GeneGO pathway analysis revealed that cell cycle arrest response was highly activated in cluster-1 and DNA repair process in cluster-2.

## RESULTS

### Peptide microarray profiling identified two clusters of pediatric AML samples

**Figure 1:**
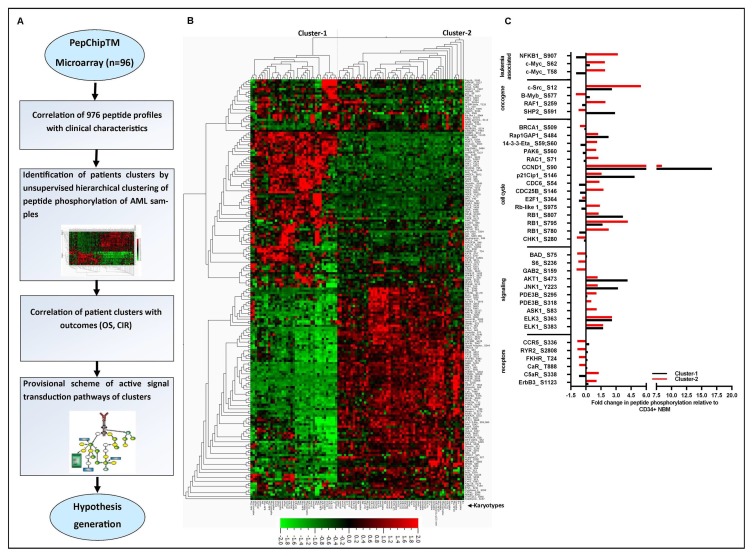
Peptide microarray profiling and its relation with patient outcome. (**A**) The overview of study design for peptide microarray profiling of pediatric AML samples to identify active signaling cascade and potential druggable targets depicted in this figure. PepChipTM microarray data of 96 AML patients were analyzed using unsupervised hierarchical clustering. Patients characteristics were correlated with clusters. Provisional scheme of the activated signal transduction pathways between two clusters was analyzed by Metacore GenoGo software. (**B**) Unsupervised hierarchical clustering of the activated peptides of the array using average linkage algorithm is presented in this figure. The heat map graphically represents the 192 activated peptides constructed from quantile normalized peptide activation intensities. Patients are significantly separated into two actual clusters by gap statistics depending on the peptide activation intensities. The patient karyotypes are marked in the bottom of the figure. Peptide activation activity is scaled so that green represents lower activation and red represents higher activation. The 192 peptides names corresponding with the phosphorylation consensus sequences of the proteins they originated from were presented in Supplementary Table 4 (**C**) Bar diagram shows the significant fold differences for a selective group of interesting categorized peptides between the AML samples of two clusters (cluster-1 and cluster-2) and CD34+ NBM controls (*n* = 4). Proteins from which the peptide sequences are derived and the explicit peptide phosphorylation sites are indicated in the graph.

Figure 1A outlines our approach to investigate the peptide microarray profiles. We hypothesized that these peptide microarray profiles contained detailed intracellular insights providing predictive signaling information. We performed unsupervised hierarchical clustering with variance 0.15 (σ/σ_max_) using average linkage algorithms. That resulted in 192/976 recorded peptides which significantly separated the samples into two main clusters (cluster-1 and cluster-2) based on gap statistics (average normalized expressions for each peptide in Figure 1B). Additionally, we tested using increased threshold (filter variance 0.25) and obtained 95 differentially activated peptides with a similar binary pattern but the CIR was not significantly different between the clusters (Supplementary Figure 2). The peptide sequences of the 192 peptides with their basic functions based on the proteins they are derived from are given in Supplementary Table 4. These 192 activated peptides belonged to proteins involved in cell cycle regulation, cell proliferation, cell differentiation, apoptosis, PI3K/AKT signaling, mitogen-activated protein kinase signaling (MAPK), metabolism regulation, transcription factors or G-protein-coupled receptors (GPCRs) signaling. It can be appreciated that general AML associated peptides (c-Myc, FOXO3A, CREB, NF-ĸβ, E2F1, C/EBP-beta, EP300, CD19) were found to be activated in these AML samples.

### Aberrant peptide microarray profiles observed for two patients cluster in comparison to CD34+ normal bone marrow (NBM)

Furthermore, we explored our analysis to investigate the aberrant peptides activated of AML samples compared with CD34+ NBM controls using the 192 differentially activated peptides. Interestingly, 161 of the total 192 activated peptides were significantly different between AML samples of both cluster 1 and cluster 2 versus CD34+ NBM controls (Supplementary Table 1). The well-known peptides are categorized and highlighted in Figure 1C. As expected, AML-associated peptide activation of c-Myc, c-Src, NFKB1 was found to be enhanced in AML especially in cluster-2 patients as compared with CD34+ NBM. Moreover, oncogene-related peptide activity of B-Raf, SHP-2 showed to be commonly upregulated in AML samples mostly in cluster-1 patients as compared with CD34+ NBM. Interestingly, we found increased peptide activation of cell cycle regulators Rb, Rb-like-1, CDC25B, CDC6, RAP1GAP1, RAC1, CCND1 and p21cip1 that showed a strong difference in absolute peptide activation intensities between AML as compared with CD34+ NBM in coexistence with a decrease in 14-3-3 eta, BRCA1, Chk1 and E2F1 peptide activity. As expected, increased AKT, MAPK related peptides ELK1, ELK3, JNK1 were highly activated in AML samples as compared with CD34+ NBM. In summary, using peptide microarray profiling, we showed the impaired activity of cell cycle regulators, PI3K and MAPK signaling pathways in AML samples as compared with CD34+ NBM samples. The detailed list of significantly upregulated and downregulated protein derived peptide activation in AML samples of two clusters is presented in Supplementary Table 1.

**Figure 2:**
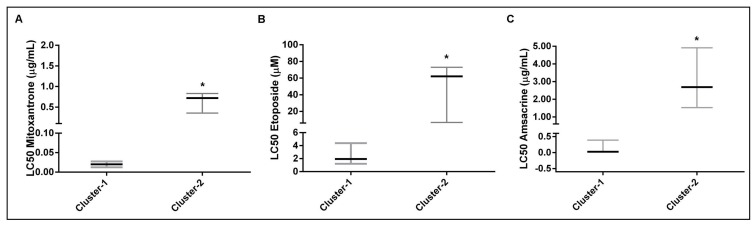
Evaluation of the chemotherapeutic drug sensitivity between AML cells of two clusters. (**A**–**C**) Differences in AML cell viability between AML cells of cluster-1 and cluster-2 samples for (A) mitoxantrone, (B) etoposide and (C) amsacrine. Cluster-1 AML cells were significantly more sensitive to all chemotherapeutic drugs (*p* < 0.01 for mitoxantrone, *p* < 0.01 for etoposide and *p* < 0.03 for amsacrine). The median LC_50_ is depicted as the horizontal solid line with interquartile range. Differences in the distribution of LC50 values were analyzed using nonparametric Mann- Whitney *U* test. *P* < 0.05 was considered statistically significant (2-tailed test).

In addition, we investigated the chemosensitivity between these two clusters to underscore the differences examined in the two clusters. As shown in Figure 2A–2C, LC 50 values are given for 10 AML samples. Cluster-2 samples showed to be significantly less sensitive to mitoxantrone (median, 31-fold, Figure 2A), etoposide (median, 31-fold, Figure 2B) and amsacrine (median, 104-fold, Figure 2C) than cluster-1 samples. The dose-dependent effect of these three drugs on cell viability between the leukemic cells of the two clusters are shown in Supplementary Figure 3A–3C. Furthermore, apoptosis assay by annexin v and propidium iodide staining showed that samples of Cluster-2 were less sensitive to mitoxantrone (0.5 μg/ml for 48 hours treatment) than Cluster-1 samples (Supplementary Figure 3D).

### Comparison of patient characteristics and outcome between two AML clusters

**Figure 3:**
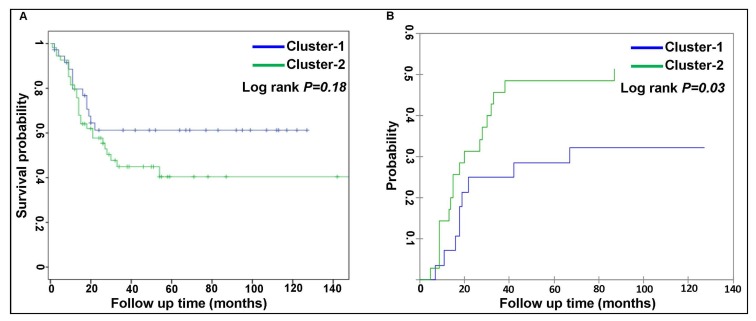
Comparison of patient outcome between two AML clusters. (**A**) Kaplan-Meier estimates for patients over time in months for the proportion of surviving patients (overall survival) between two clusters (non-significant, *p* = 0.18) and (**B**) Significant differences of cumulative incidence of relapse (CIR) is presented between the patients of two clusters (*p* < 0.03).

No correlation between individual peptide activation levels and clinical characteristics (e.g. age, sex, FAB classification, karyotypes, blast percentage, white blood cell count, red blood cell count and platelet count) with multiple testing corrections was observed. The patient karyotypes of two clusters are shown in Supplementary Table 2. No significant differences in patient characteristics (e.g. age, sex, blasts percentage, white blood cell count, platelet count, frequencies of relapse, death or complete response and sample sources of either peripheral blood or bone marrow) were found between the two clusters. The median time to complete remission between the two clusters (37.5 ± 5.6 days for cluster-1 vs 40.5 ± 2.5 days for cluster-2) was also not significantly different. Only red blood cell count was significantly lower in the patients in cluster-2 (4 × 10^9^/L) compared to cluster-1 (5 × 10^9^/L) (Table 1A). Additionally, frequencies of the recurrent genetic abnormalities according to WHO classification [17] and mutational profiles were similar between the two clusters (Table 1B). Only FLT3-ITD mutation frequency was higher in cluster-2 patients than cluster-1 although it was not significant. In our data set, mutated NPM1 with or without FLT3-ITD was not found as definite entity. The overall survival was also not statistically significantly different between the two clusters (log-rank test, Figure 3A) whereas CIR of cluster-2 (47%) was significantly higher than of cluster-1 (26%) (χ^2^ test, Figure 3B).

**Table 1:** Patient characteristics **(A)**

Characteristics (*n* = 96)	All patients (mean ± SD)/(no/96)	Patient in Cluster-1 (*n* = 39)	Patient in Cluster-2 (*n* = 57)	Cluster-1 vs Cluster-2 (*p*-value)
Age (years)	8.3 ± 4.7	8.2 ± 4.7	8.4 ± 4.8	0.75
Gender (%)				
Male	65.6	22	41	0.16
Female	31.3	15	15	
Unknown	3.1			
BM Blast (%) (mean ± SD)	71.7 ± 20	72.6 ± 20.1	71.1 ± 20.1	0.72
PB Blast (%) (mean ± SD)	59.3 ± 25.4	59 ± 26.9	59.4 ± 24.6	0.92
WBC, **×**10^9^/L (mean ± SD)	29.9 ± 22.1	29.6 ± 23	30.1 ± 21.7	0.69
RBC, **×**10^9^/L (mean ± SD)	4.4 ± 1.4	4.9 ± 1.3	4.1 ± 1.3	0.01^*^
PLT, **×**10^7^/L (mean ± SD)	37.9 ± 30	37.1 ± 27.8	38.5 ± 24.6	0.51
Relapse (no/96)	36	10	26	0.03^*^
Death (no/96)	43	15	28	0.27
Complete response (no/96)	64	28	36	0.29

Results were considered statistically significant when *p*-values were <0.05. Associations between two clusters and patient characteristics were assessed in SPSS (Statistical *Package* for the Social Sciences). Mann-Whitney *U* test (for continuous variables) was used to assess the difference between the two clusters.

**(B)**

**Table:** 

Characteristics (*n* = 96)	Number of patients
French-American-British classification (no/96)	
M0	8
M1	12
M2	22
M3	2
M4	20
M5	26
M6	1
unknown	5
WHO-classification (no/96)	
AML with recurrent genetic abnormalities (*n* = 27)	
t(8;21)/RUNX1-RUNX1T1	13
inv(16)/CBFB-MYH11	8
t(15;17), PML-RARA	1
t(9;11)/MLLT3-MLL	5
• AML otherwise not specified	37
• Molecular Genetic Alternation (*n* = 32)	
NPM1	1
CEBPA	3
FLT3-ITD	9
N/K-RAS	9
c-KIT	7
WT1	3

### Delineation of signal transduction networks of two AML clusters

To understand the different signaling dynamics activated in the two clusters, we performed pathway analysis of highly activated peptides for both cluster-1 and cluster-2 separately. Metacore GeneGO pathway analysis was used to identify the top ranked most significantly altered pathways using enrichment analysis. Top 20 pathways for proteins of corresponding peptides upregulated in both cluster-1 and cluster-2 in GeneGo are presented in Supplementary Table 3. Pathways selectively upregulated in cluster-1 were: immune response related signaling pathways, DNA damage response, cell cycle arrest, p53 dependent apoptosis and melanoma progression. Pathways uniquely upregulated in cluster-2 included: receptors mediated signaling pathways (specifically via IGF-1receptor, C5a receptor, Delta-type opioid receptor, A1 receptor, A2 receptor and G-protein coupled receptors), downstream of AKT signaling pathways, c-Kit ligand signaling pathways, apoptosis inhibition and DNA repair signaling pathways. The most interesting key difference between the pathways of the two clusters was related to proteins being involved in cell cycle arrest, cell cycle checkpoints, cell cycle transition, DNA repair and DNA replication. Therefore, we built a protein-protein interaction network using peptides that are involved in cell cycle regulation.

**Figure 4:**
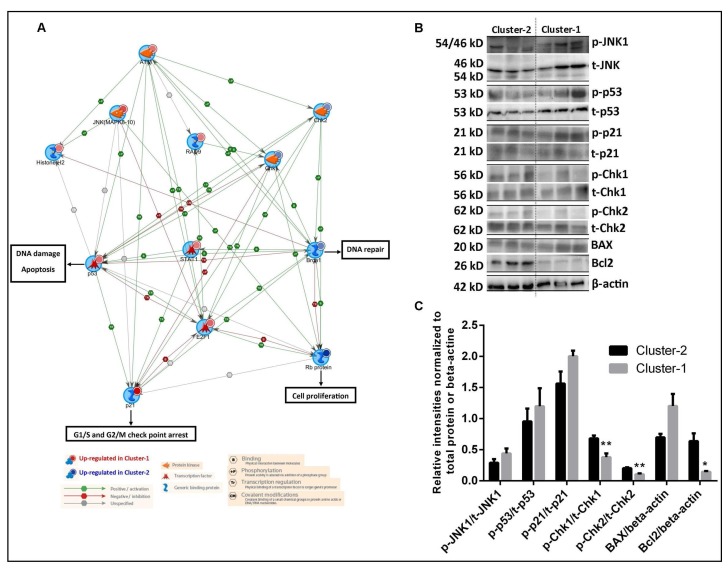
Delineation of signal transduction networks and determination of chemosensitivity of two AML clusters. (**A**) Metacore GeneGo graphic illustration of the peptides involved in cell cycle regulation between two clusters. Network analysis was performed using peptides involved in the cell cycle regulatory pathway. The differentially activated peptides identified from two clusters are represented by circles (red colored circle denoted those peptides were upregulated in cluster-1 and blue colored circle denoted those peptides were upregulated in cluster-2). Protein objectives are represented by various shapes and colors depending on their functional annotations and colors of the lines with the indication of the functional interaction with other peptides are shown in the bottom of the Figure. (**B**) Immunoblots confirmation of the peptide activation array results using leukemic blasts of three leukemic patients for each cluster. Immunoblots showing the variation in phosphorylation levels of JNK1, p53, p21, Chk1 and Chk2 protein and for BAX and Bcl2 total protein between the AML patients of two clusters. (**C**) The quantification of the western blot analysis is presented as a bar diagram. Phosphorylation of JNK1, p53, p21, Chk1 and Chk2 proteins were normalized with their total protein levels. For BAX and Bcl2 protein levels were normalized against β-actin. ^*^Significant differences between two individual groups were determined by using Student’s *t*-test, *p* < 0.05.

This network analysis suggested that the cell cycle axis within DNA damage response was highly activated in cluster-1 patients as p53 and p21 peptides were intense on the microarray (Figure 4A). In cluster-1, it seems that highly phosphorylated p53 may contribute to G1/S and G2/M checkpoint arrest via induction of p21. Analyzing ATM and γ-H2AX in our array data, both peptides were relatively intensely activated in cluster-1 patients based on absolute intensities (median intensity for ATM, 264 vs 16 and for H2AX, 98 vs 43 in cluster-1 vs cluster-2 respectively). Phosphorylated p53 can be activated by DNA damage-inducible kinase, such as ATM and also by H2AX and promote apoptosis. In addition, higher E2F1 phosphorylation presumably catalyzed by ATM kinase was found in cluster-1 and this phosphorylation may induce p53 phosphorylation and apoptosis in cluster-1.

In cluster-2, higher intensities of BRCA1, Chk1 and Chk2 were found. BRCA1 is known to be phosphorylated by Chk1 and Chk2 phosphorylation. Highly phosphorylated BRCA1 could participate in DNA repair mechanism and probably facilitate higher DNA replication process of cluster-2 AML cells. In addition, it could be hypothesized that faster cell cycle progression is promoted by higher phosphorylation of Rb and lower E2F1 phosphorylation in cluster-2 patients. These data suggest that the AML samples in cluster-1 have elevated p53 signaling possibly leading to apoptosis while AML samples in cluster-2 have elevated signaling reflected in DNA damage tolerance and repair.

To confirm peptide microarray results of the cell cycle regulatory process, western blot analyses were conducted for phosphorylation status and total amounts of JNK1, p53, p21, Chk1, Chk2 using leukemic blasts of three leukemic patients for each cluster (Figure 4B, 4C). It can be appreciated that p-JNK1, p-p53 and p-p21 were slightly elevated in cluster-1 compared to cluster-2 patients (non-significant,) and p-Chk1 and p-Chk2 levels were found to be significantly higher in cluster-2 compared to cluster-1 patients as expected. Furthermore, in cluster-1 patients, we hypothesized that activated p53 may induce apoptosis signals. Within the context of higher p53 activation in cluster-1, a tendency of higher pro-apoptotic BAX levels (non-significant) and significantly lower levels of anti-apoptotic Bcl2 were found in cluster-1 patients compared to cluster-2 suggesting that AML cells of cluster-1 patients are more prone to apoptosis.

## DISCUSSION

This study has demonstrated that peptide microarray profiling of AML samples provides significant details about AML signaling and identified two clusters associated with DNA damage response and repair system, resulting in significantly different CIR. The present study shows the power of high throughput peptide microarrays for the first time in a larger number of pediatric AML patients.

Our previous studies have shown the feasibility of peptide microarray technology to develop treatment possibilities with specific kinase inhibitors for pediatric brain tumor, acute lymphoblastic leukemia and subsets of AML patients [16, 18, 19, 20]. Although peptide microarray screens provide an excellent opportunity to simultaneously screen for phosphorylation of the kinome domain on a huge number of peptide substrates, this technique also has some known limitations. *In vitro* peptide microarray profiling may reveal phosphorylation of specific peptides which may not occur *in vivo* since the tertiary structure of phosphorylation sites and the spatiotemporal regulatory mechanisms are lost [21]. Therefore, concomitant protein analyses as well as network analyses which built pathways of intensely activated peptides together, attenuates this disadvantage. Another limitation in our study is that with the technique of peptide microarrays we are not yet able to use single cell analysis on this platform. Therefore, we were not able to take different AML clones into account and our present data focused on the comprehensive signal transduction pathways in whole AML cell lysates. In this era, each year multiple new specific kinase inhibitors are developed and brought to the clinical setting. Moreover, treatment with a single kinase inhibitor in AML has not been successful often due to bypass or escape routes in AML cells that we have shown recently by developing a novel combination therapy for MLL-rearranged AML [21]. With MEK inhibition AML cells developed alternative escape routes allowing survival via extreme induction of VEGFR-2, while the combined inhibition of both MEK and VEGFR-2 limited cellular recovery was found via decreased AKT and mTOR activity. Another study explained that inhibiting mutations often showed the lack of sensitivity in *in vitro* culture and organoids [22]. Therefore, it is essential to elucidate more signaling insights in the cancer cell. Our current peptide microarray profiling resulted in several observations.

At first, this study characterized the deregulated signaling pathways in pediatric AML. The peptide microarray profiles provided a comprehensive insight into signaling pathways active in pediatric AML patients. As expected, we found activation of proteins belonging to cell cycle regulation, cell proliferation, cell differentiation, apoptosis, PI3K/AKT signaling, MAPK signaling along with the regulators of metabolism, transcription factors and GPCRs signaling regulators in pediatric AML. Moreover, AML-associated peptides (c-Myc, FOXO3A, CREB, NF-ĸβ, E2F1, C/EBP-beta, EP300, CD19) were found to be activated in our array. This finding underscores our previous observation in leukemia that CREB, NF-ĸβ, E2F1 and EP300 were highly phosphorylated in MLL-rearranged AML [16]. Other studies also showed the activation of FOXO3A, C/EBP-beta, Btk, LKB1, CD19 in AML as we have seen in the present study that FOXO3A and LKB1 peptides were highly activated in cluster-2 patients and C/EBP-beta, CD19, Btk were highly activated in cluster-1 patients which suggests importance of this approach to identify targeting therapies for individual patients [23–28]. To date, however, nothing is known about the phosphorylation activity status of GPCRs in AML, therefore, these receptors could be interesting targets for further studies in the context of leukemia. Thus, it can be appreciated that, for the first time we showed the activated signaling pathways in pediatric AML using this peptide microarray.

Secondly, by unsupervised hierarchical clustering, two clusters of AML samples were identified, without the knowledge of prior class definitions and independent of patient characteristics e.g. blast percentage, WBC counts, age, sex and karyotypes but related to CIR. Moreover, *in vitro*, cluster-2 samples were more resistant to chemotherapeutic drugs than cluster-1 samples. Dissecting the peptides activation by bioinformatics analyses and subjecting the activated peptides to pathway analysis yields direct insights into signaling differences into the two clusters. It is tempting to hypothesize that the lower CIR found in cluster-1 AML samples is related to some remaining functionality of p53 and p21 in driving the cell towards cell cycle arrest, followed by apoptosis as shown in the low Bcl2 and high BAX protein levels. Mutations in p53 are rare in pediatric AML (approximately 1%) and correlate with worse outcome [29]. Previously, we showed that p53 can be activated and be functional in AML inducing cell cycle arrest [30]. Activation of p53 is known to result in the induction of p21 which further results in Rb-mediated E2F1 inhibition to prevent cell cycling. Independently of p53, p21 activation itself can inhibit CDK2 and prevent cells from further cell proliferation. Although E2F1 is known as a master regulator of cell proliferation, there is emerging evidence that its role is only visible in cancer cells and not in normal cell cycling [31]. It is known that E2F1 activity is also regulated by post-translational mechanism besides its regulation by Rb proteins. For example, it was shown previously that, increased E2F1 phosphorylation may phosphorylate p53 and induce apoptosis by increasing E2F1-half-life by inhibiting Rb protein [32, 33].

In cluster-2 samples, high BRCA1 activation may facilitate DNA repair mechanism as well as the phosphorylation of Rb. BRCA1 plays a pivotal role in the repair of DNA damage, especially induced following chemotherapy and ionizing radiation. There are only limited data on the role of BRCA1 activation in leukemia, higher BRCA1 expression was found in resistant AML sample [34, 35]. Phosphorylated BRCA1 is known to interact with different proteins e.g. the MRN complex (RAD50/MRE11/NBS1) and with BRCA2 and to facilitate repair of DNA-double strand breaks by homologous recombination [36]. Inactivation of Rb-protein by phosphorylation may promote faster cell proliferation of AML cells. Rb is well known by its tumor suppressor property, generally attributed to its ability to bind to E2F family members. Released E2F from their binding with Rb are able to facilitate transcription of cell cycle genes. Previous studies suggested that Rb does not only induce its suppressor function through a direct interaction with E2F, but is capable of exerting broader effects on transcriptional controls and chromatin structure [37, 38]. So, based on our results, we hypothesized that AML samples belonging to cluster-2 were characterized by a pattern of high BRCA1 phosphorylation and higher Rb phosphorylation (inactive) that may promote more cell proliferation of cluster-2 AML cells. The increased ability to repair DNA damage is reflected in an increased incidence of relapse in cluster-2 AML samples compared to cluster-1 AML cells.

In conclusion, this study demonstrates the feasibility and strength of this peptide microarray array platform in a large group of AML samples. Results elucidated deeper insights in the active signaling pathways, its high interconnections, as well as the essential role cell cycle regulation plays in the observed difference in the cumulative incidence of relapse. And so it is tempting to explore whether the use of cell cycle inhibitors focusing on DNA damage response and DNA repair pathways might be beneficial new additives to AML treatment schedules.

## MATERIALS AND METHODS

### Patient population, samples collection and processing

This study used a total of 100 primary blood or bone marrow samples of newly diagnosed AML patients. Patients were treated according to Dutch Childhood Oncology Group (DCOG). After obtaining written informed consent, primary AML samples at diagnosis were collected from pediatric patients in accordance with the declaration of Helsinki and the study was approved by all participating review boards. Mononuclear cells were separated by Lymphoprep density gradient (Nycomed, Oslo, Norway), and cryopreserved in liquid nitrogen until use. The cryopreserved leukemia cells were thawed rapidly at 37° C and diluted in a 6 ml volume of newborn calf serum, as described previously [16]. The remaining blast cell population contained >95% leukemia cells with PI staining, as shown in our previous study and is referred to hereafter as leukemia cells [16]. The associated patient characteristics and also based on FAB and WHO classifications are described in Table 1A and 1B.

### Peptide microarray array and data analysis

The peptide microarray profiles of AML samples (*n* = 96) and CD34+ normal bone marrow (NBM) samples (*n* = 4) were determined using the PepChipTM Kinomics microarray system (Pepscan, Lelystad, The Netherlands) as described previously [39]. Briefly, the peptide array contains 976 different kinase peptide-substrates, each spotted as triplicates. The protein-derived peptide sequences contain phosphorylation sites that can be used as substrates for kinases active in the samples. The assay readout is the net sum of phosphorylation at each peptide, whether acted on by one kinase, or several different kinases. The patient samples (0.5 × 10^6^ AML cells) were lysed in 100 μl of cell lysis buffer (20 mM Tris-HCl, pH 7.5, 150 mM NaCl, 1 mM Na_2_EDTA, 1 mM EGTA, 1% Triton X-100, 2.5 mM sodium pyrophosphate, 1 mM MgCl_2_, 1 mM β-glycerophosphate, 1 mM Na_3_VO_4_, 1 mM NaF, 1 mg/ml leupeptin, 1 mg/ml aprotinin, 1 mM PMSF). Peptide array incubation mix was produced by adding 10 ml of filter-cleared activation mix (50% glycerol, 50 mM [γ-^33^P] ATP, 0.05% v/v Brij-35, 0.25 mg/ml bovine serum albumin, [γ-^33^P] ATP (1000 kBq)). Next, the peptide array mix and sample lysate were added onto the chip and incubated at 37° C in a humidified stove for 90 min. Subsequently, the peptide chip was washed twice with Tris-buffered saline with Tween, twice in 2M NaCl, and twice in demineralized H_2_O and then air-dried at 37° C.

The chips were exposed to a phosphoimager plate for 72 hours, and the density of the spots was measured and analyzed with array software (ScanAlyze, version 2.50, Eisen Software). Using grid tools from ScanAlyze software, spot density and individual background intensities were analyzed. An overview of the data analysis procedure is provided in Supplementary Figure 1. First, background signal intensities were subtracted from spot intensities and then data were quantile normalized. A pre-filtering step was performed by calculating Pearson correlation coefficient over the triplicates. Samples were excluded (4/100) from the analysis when the Pearson’s Correlation coefficient (*r*^2^) of triplicates was below 0.6. For further analysis medians from the triplicates were used. Quantile normalized data were analyzed using Qlucore Qmics Explorer version 3 (Qlucore AB, Lund, Sweden). In order to identify differentially activated peptides and patients clusters, peptides with variance 0.15 (σ/σ_max_) were selected for further analysis [40, 41]. That resulted in 192/976 recorded peptides as the lowest number of peptides making a difference in outcome. Subsequently, unsupervised hierarchical clustering analysis with average linkage algorithm was performed and graphical representation of the results of clustering was done with this software. The estimated number of clusters was counted by gap statistics [42].

### Leukemic cell viability assay

The WST-1 assay was performed to assess the leukemic cell viability of primary leukemic samples after treated with chemotherapeutic drugs as described previously [16]. Briefly, this assay was performed in sextuple according to manufacturer’s protocol (Roche). AML cells were seeded at a density of 0.1–1 × 10^5^ cells per 100 μl/well in a 96 wells plate in RPMI medium supplemented with 10% fetal calf serum with and without chemotherapeutic drugs (range of concentration), mitoxantrone (0.01–10 μg/mL, Sandoz BV, The Netherlands), etoposide (0.01–100 μM, Pharmachemie BV, The Netherlands) and amsacrine (0.01–10 μg/mL, ProStrakan Pharma, The Netherlands). These concentration ranges were determined empirically to obtain the largest number of evaluable dose-response curves in AML and allowing the calculation of LC50 values. The mitochondrial activity of AML cell was measured after 48 h using a microplate reader at 450 nm (Benchmark; Bio-Rad Laboratories). Cell survival percentages (viability) are determined relative to non-treated cells.

The cell viability data is presented by calculating the LC50 for leukemic cells with the following formula:

$$\begin{array}{l} LC50=\frac{[\%\;viability>50\%]-50}{\left[\%\;viability>50\%]-[\%\;viability<50\%\right]}\times (drug\;conc.\;when\;viability<50\%\\ -drug\;conc.\;when\;viability>50\%)\;+\;(drug\;conc.\;when\;viability>50\%) \end{array}$$

Differences in the distribution of LC50 values were analyzed using nonparametric Mann-Whitney *U* test. *P* < 0.05 was considered statistically significant (2-tailed test). The difference in median LC50 values for a specific drug between 2 groups of patients was presented in the graph.

### Pathway analysis

A system biology pathway analysis tool, MetaCore^TM^ (GeneGo Inc., St Joseph, MI, USA), based on a manually curated database of known molecular interactions, pathways, and processes, was used to explore the signaling pathways of the 192 differentially activated peptides. The activated peptides of the two different clusters were compared to score and rank entities in pathways most relevant for these 192 peptides. In MetaCoreTM, the statistical significance (*p*-value) is calculated using a hypergeometric distribution. Additionally, the GeneGo pathway analysis platform was used to generate a protein-protein interactional network, based on evidence in literature [39].

### Western blot

1 × 10^6^ cells were lysed in laemmli sample buffer (Bio-Rad Laboratories, Veenendaal, The Netherlands). Proteins were separated by SDS-polyacrylamide gel electrophoresis, and transported to nitrocellulose membranes as described previously [21]. First, the membranes were incubated overnight with primary antibodies for p-JNK1, t-JNK1, p-p53, t-p21, p-Chk1, p-Chk2, Bcl2, BAX and β-actin (Cell Signaling), t-p53 (BD Biosciences) and p-p21, t-Chk1, t-Chk2 (Santa Cruz) followed by 1 h incubation with HRP-conjugated secondary antibodies (DAKO Cytomation). Protein bands were visualized by chemiluminescence, using ChemiDoc MP System (Bio-rad laboratories).

### Statistics

One way ANOVA was used for multiple comparisons of peptide activation between AML patients of cluster-1 and cluster-2 and CD34+ NBM samples and *p*-values were presented with Bonferroni corrections. The Kaplan-Meier method was used to generate the survival curves between two clusters (log-rank, χ^2^ test). Cumulative incidence of relapse (CIR) which is the measure of relapse frequency during the follow-up time between two clusters was tested using Pearson Chi-Square test. Results were considered statistically significant when *p*-values were < 0.05. Associations between two clusters and patient characteristics were assessed in SPSS (Statistical *Package* for the Social Sciences). Mann-Whitney *U* test (for continuous variables) and Pearson Chi-Square test (for categorical variables) were used to assess the difference between the two clusters.

## SUPPLEMENTARY MATERIALS








