# Supplementary material for: Peptide microarray of pediatric acute myeloid leukemia is related to relapse and reveals involvement of DNA damage response and repair
Source: Oncotarget. 2019 Jul 23;10(45):4679–90. doi: 10.18632/oncotarget.27086 (PMC6659796; doi:10.18632/oncotarget.27086)
Supplement: Supplementary file 1 [file oncotarget-10-4679-s001.pdf]

Peptide microarray of pediatric acute myeloid leukemia is related to relapse and reveals involvement of DNA damage response and repair

SUPPLEMENTARY MATERIALS

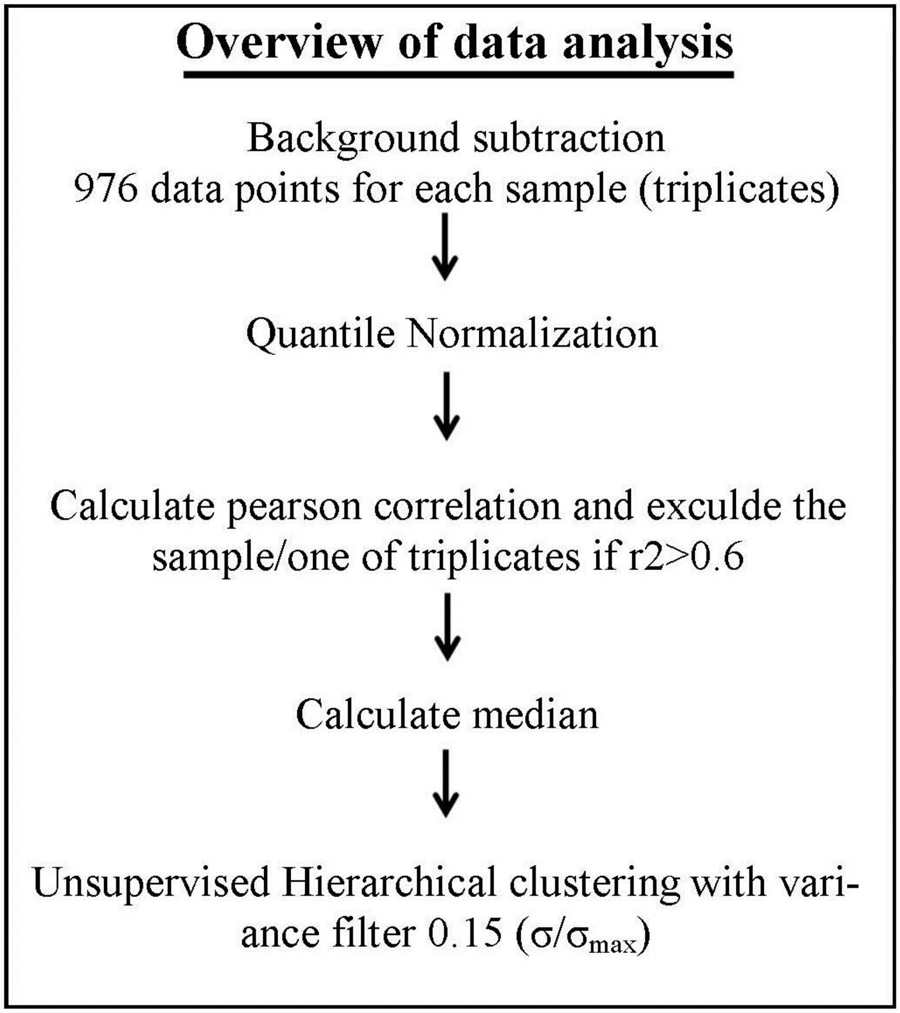

Supplementary Figure 1: Overview of data analysis.

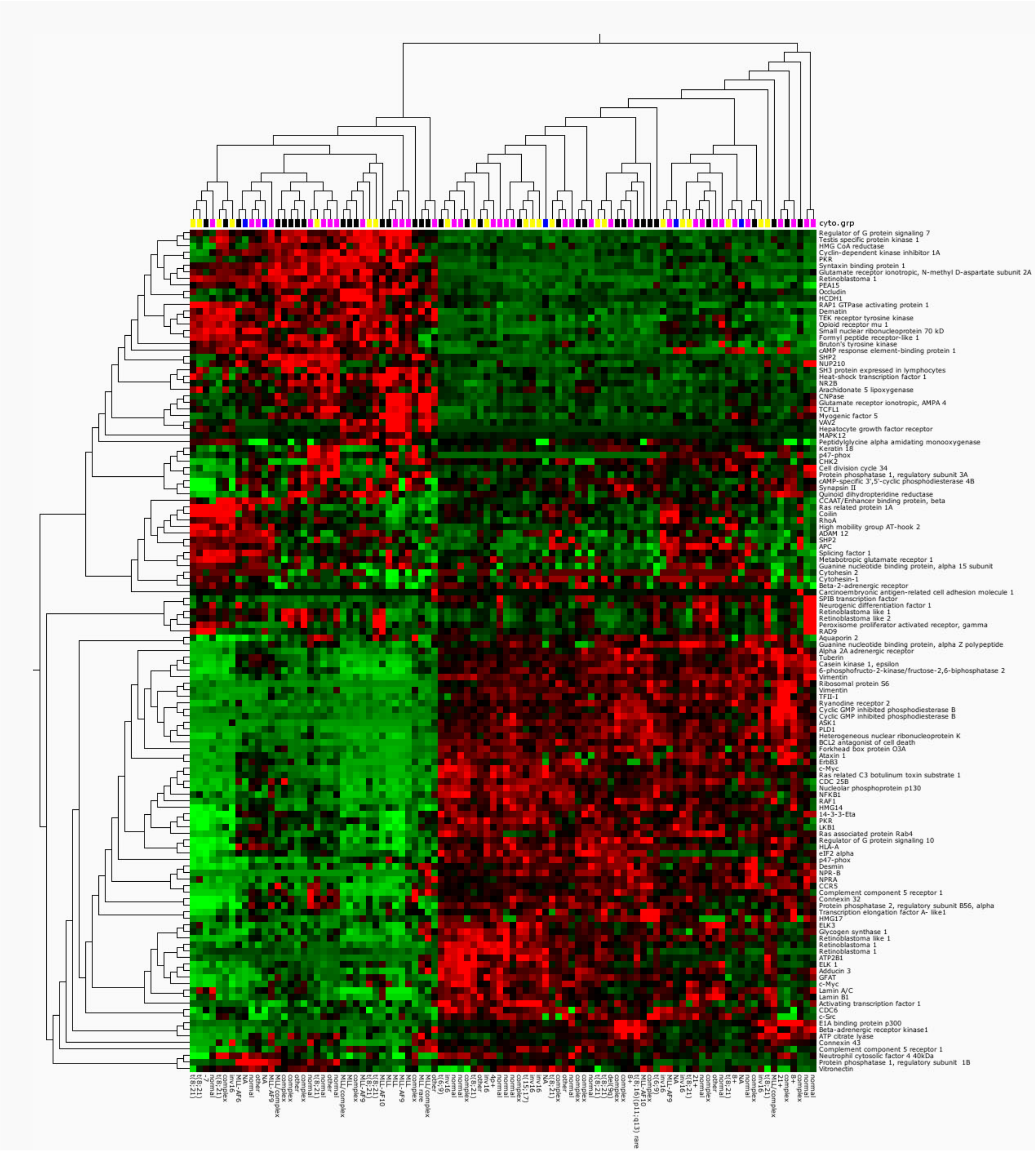

Supplementary Figure 2: Unsupervised hierarchical clustering of the activated peptides of the array using average linkage algorithm with increased threshold (filter variance 0.25). A total 95 differentially activated peptides were obtained with a similar binary pattern but the CIR was not significantly different between the clusters.

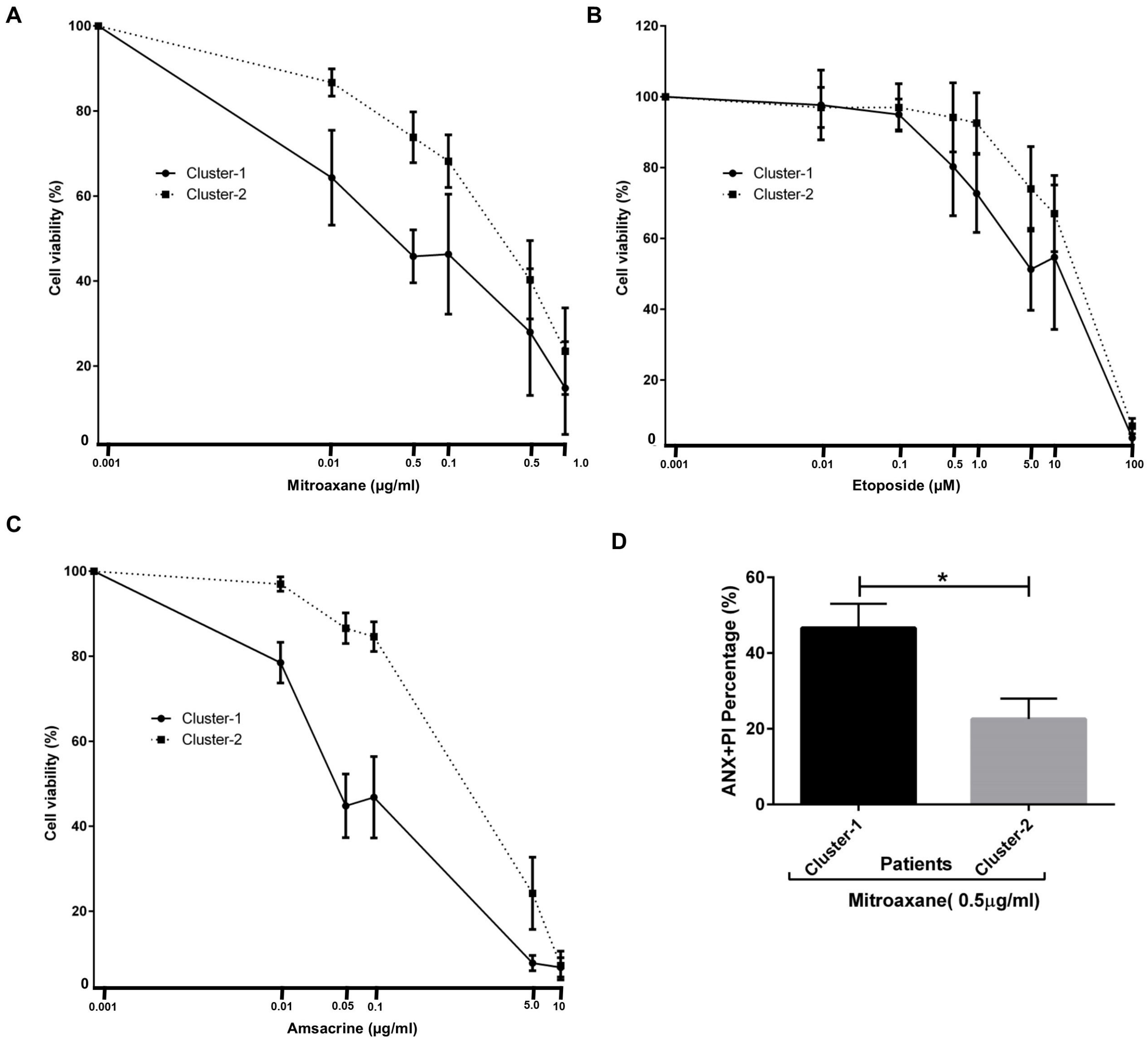

Supplementary Figure 3: (**A–C**) Dose-dependent viability reduction in primary AML cells of cluster-1 and cluster-2 patients exposed to either Mitroaxane, Etoposide and Amsacrine. **(D)** Percentage of apoptosis for the samples of Cluster-1 showed significantly higher in compare to Cluster-2 when AML cells were treated with Mitroxantrone.

Supplementary Table 1: List of significantly upregulated and downregulated protein derived peptide activation in AML samples of two clusters (*n* = 96) as compared to CD34+ NBM (*n* = 4). See Supplementary_Table_1

Supplementary Table 2: Different patient karyotypes in two clusters

Karyotypes	Cluster-1 % (*n* = 39)	Cluster-2 (*n* = 57)
Complex	13% (5)	23% (13)
Normal	15% (6)	21% (12)
MLL	31% (12)	7% (4)
Inv(16)	8% (3)	9% (5)
t(8;21)	15% (6)	12% (7)
Other	18% (7)	28% (16)

Supplementary Table 3: The 20 most significantly altered pathways of 192 activated peptides, upregulated peptides in cluster-1 and upregulated peptides in cluster-2. See Supplementary_Table_3

Supplementary Table 4: List of 192 peptides that were highly activated in cluster-1 (C1) and cluster-2 (C2) with peptide name with p-site, sequence and with their functions. Peptides are sorted based on the functions and presented. See Supplementary_Table_4
