# Supplementary material for: Peptide microarray of pediatric acute myeloid leukemia is related to relapse and reveals involvement of DNA damage response and repair
Source: Oncotarget. 2019 Jul 23;10(45):4679–90. doi: 10.18632/oncotarget.27086 (PMC6659796; doi:10.18632/oncotarget.27086)
Supplement: Supplementary file 2 [file oncotarget-10-4679-s002.docx]

**Supplementary Table 1:** List of significantly upregulated and downregulated protein derived peptide activation in AML samples of two clusters (n=96) as compared to CD34+ NBM (n=4).

| **Significantly upregulated in both clusters** | |
| --- | --- |
| ***Peptide name*** | ***Bonferroni P-value*** |
| RB1_ S807 | 7.31E-34 |
| STXBP1_ S313 | 1.06E-28 |
| AKT1_ S473 | 1.01E-24 |
| NMDAR2A_ Y1105 | 6.88E-23 |
| PEA15_ S104 | 4.73E-22 |
| TEK_ Y992 | 1.05E-21 |
| Dematin_ S403 | 1.96E-21 |
| FPR2_ S236 | 3.12E-21 |
| Rap1GAP1_ S484 | 1.17E-20 |
| SH2D3A_ Y95 | 9.21E-18 |
| p21Cip1_ S146 | 2.08E-17 |
| HSF1_ S230 | 3.78E-15 |
| ELK3_ S363 | 3.71E-13 |
| VRK1_ T305 | 8.5E-13 |
| snRNP70_ S137 | 5.21E-12 |
| RGS2_ S46 | 1.85E-11 |
| TPH1_ S58 | 1.73E-10 |
| JNK1_ Y223 | 5.86E-10 |
| Lamin A/C_ S22 | 9.76E-10 |
| c-Src_ S12 | 1.36E-09 |
| GluR4_ S862 | 2.14E-09 |
| NUP210_ S1881 | 1.08E-08 |
| RB1_ S795 | 2.58E-08 |
| LEPR_ Y986 | 6.42E-08 |
| ELK3_ S357 | 8.61E-08 |
| MYF5_ S130 | 3.65E-07 |
| HCDH1_ S151 | 6.01E-07 |
| Neurogranin_ S36 | 1.48E-06 |
| 5-LO_ S271 | 9.93E-06 |
| CCND1_ S90 | 1.15E-05 |
| p90RSK_ S227 | 0.000343 |
| KRT8_ S74 | 0.000384 |
| ADAM 12_ Y907 | 0.000437 |
| HMGA2_ S59 | 0.002379 |
| NEUROD1_ S274 | 0.003749 |
| DARPP-32 _ T34 | 0.00379 |
| KRT8_ S431 | 0.013304 |
| C/EBP-beta_ T235 | 0.016126 |
| SMAD2_ T220 | 0.033824 |
| APLP2_ T723 | 0.039407 |
| SHP2_ Y542 | 0.040034 |
|  |  |
| **Significantly downregulated in both clusters** | |
| ***Peptide name*** | ***Bonferroni P-value*** |
| GAB2_ S159 | 6.91E-37 |
| hnRNP K_ S302 | 3.26E-34 |
| S6_ S236 | 1.05E-31 |
| BAD_ S75 | 1.3E-31 |
| CRMP-2_ T555 | 2.85E-29 |
| NFAT1_ S268 | 2.88E-28 |
| RYR2_ S2808 | 1.06E-27 |
| Vimentin_ S26 | 1.18E-25 |
| CTNNG_ S2849 | 1.81E-25 |
| TFII-I_ S412 | 3.36E-25 |
| FOXO3A_ T32 | 5.19E-25 |
| CaR_ T888 | 1.01E-23 |
| PFKFB3_ S483 | 1.11E-23 |
| PDE3B_ S318 | 5.47E-23 |
| Ataxin-1_ S776 | 1.02E-22 |
| DDX5_ S557 | 1.32E-21 |
| BRCA1_ S509 | 1.79E-21 |
| PFKFB3_ T475 | 2.7E-20 |
| TSC2_ S939 | 2.73E-20 |
| B-Myb_ S577 | 5.53E-20 |
| CK1E_ S323 | 1.06E-19 |
| FKHR_ T24 | 1.33E-19 |
| ADRA2A_ S232 | 4.3E-18 |
| RGS10_ S176 | 9.42E-18 |
| Calpain-1_ T80 | 1.35E-17 |
| Opioid receptor_ S344 | 3.24E-16 |
| CHK1_ S280 | 4.16E-16 |
| HLA-A_ S337 | 5.97E-16 |
| CREB_ S133 | 1.04E-14 |
| CCR5_ S336 | 1.43E-14 |
| EP300_ S89 | 6.3E-11 |
| G-alpha(Z)_ S27 | 6.37E-11 |
| Desmin_ S12 | 7.32E-11 |
| Btk_ S180 | 7.43E-11 |
| NPRA_ S538 | 2.66E-10 |
| PPP2R5A_ S28 | 2.69E-10 |
| TFII-I_ S668 | 3.62E-10 |
| WIP_ S488 | 5.87E-09 |
| Cx32_ S233 | 3.25E-08 |
| PIGR_ S637 | 2.19E-07 |
| TCFL1_ S41 | 3.79E-07 |
| PKN_ S374 | 1.22E-06 |
| AQP2_ S256 | 1.38E-05 |
| CYTH1_ S394 | 6.17E-05 |
| p40Phox_ T154 | 0.000199 |
| E2F1_ S364 | 0.000464 |
| p47-phox_ S304 | 0.000662 |
| HSF1_ S303 | 0.001226 |
| ACLY_ S450 | 0.00487 |
| Cytohesin 2_ S392 | 0.005914 |
| ADRB2_ S262 | 0.006663 |
| p53_ S9 | 0.011571 |
| 5-HT(2C)_ S456 | 0.013383 |
| Cx43_ S367 | 0.035312 |
| Telethonin_ S157 | 0.044054 |
|  |  |
| **Significantly upregulated in cluster 1** | |
| ***Peptide name*** | ***Bonferroni P-value*** |
| RGS7_ S434 | 1.72E-23 |
| TESK1_ S220 | 1.16E-18 |
| MOR-1_ S268 | 6.24E-17 |
| LRP1_ Y4507 | 1.67E-13 |
| PKR_ T451 | 3.81E-12 |
| SHP2_ S591 | 5.45E-11 |
| TRPV4_ Y253 | 1.26E-10 |
| Occludin_ S340 | 5.06E-10 |
| NR2B_ S1303 | 1.3E-09 |
| CD19_ Y409 | 1.9E-09 |
| CNPase_ S9 | 4.98E-08 |
| BMX_ Y40 | 1.21E-07 |
| HMGCR_ S872 | 2.42E-07 |
| HGFR_ Y1235 | 2.74E-07 |
| p53_ S20 | 7.19E-06 |
| MAP2_ S1679 | 2.92E-05 |
| SASH3_ S27 | 6.3E-05 |
| VAV2_ Y172 | 0.000153 |
| SF1_ S20 | 0.000538 |
| Fascin 1_ S38 | 0.012023 |
| MAPK12_ Y185 | 0.027583 |
| mGluR1_ T695 | 0.028328 |
|  |  |
| **Significantly upregulated in cluster 2** | |
| ***Peptide name*** | ***Bonferroni P-value*** |
| ASK1_ S83 | 7.48E-35 |
| PLD1_ S561 | 8.62E-33 |
| Vimentin_ S72 | 3.42E-32 |
| RAC1_ S71 | 6.13E-27 |
| NPR-B_ S526 | 2.79E-25 |
| NOLC1_ S623 | 2.21E-24 |
| c-Myc_ S62 | 1.19E-23 |
| RAF1_ S259 | 9.15E-22 |
| KEL_ S63 | 1.7E-21 |
| HMG14_ S7 | 3.31E-20 |
| PDE3B_ S295 | 7.88E-20 |
| Rab4_ S204 | 6.77E-18 |
| PTPRA_ S189 | 2.97E-17 |
| p47-phox_ S320 | 8.35E-17 |
| CACNB2_ S478 | 1.02E-16 |
| NFKB1_ S907 | 1.13E-15 |
| PAK6_ S560 | 1.41E-15 |
| RB1_ S780 | 2.04E-15 |
| ADD3_ S693 | 2.55E-15 |
| PKR_ S242 | 1.39E-14 |
| CDC25B_ S146 | 1.45E-14 |
| ErbB3_ S1123 | 1.72E-14 |
| GFAT_ S205 | 1.77E-14 |
| LKB1_ S424 | 1.94E-14 |
| Rb-like 1_ S975 | 1.99E-14 |
| LMNB1_ S23 | 7.87E-14 |
| 14-3-3-Eta_ S59;S60 | 1.58E-13 |
| CD44_ S291 | 1.6E-13 |
| TOP2A_ S1213 | 7.56E-12 |
| CDC6_ S54 | 1.64E-11 |
| GYS1_ S645 | 2.82E-11 |
| Lamin B1_ S395 | 3.1E-11 |
| ATP2B1_ S1178 | 1.32E-10 |
| eIF2-alpha_ S52 | 2.53E-10 |
| SIIR_ S37 | 7.27E-10 |
| ATF-1_ S63 | 1.51E-09 |
| c-Myc_ T58 | 6.81E-09 |
| ADRBK1_ S685 | 1.39E-08 |
| ELK1_ S383 | 1.94E-08 |
| HMG17_ S25 | 2.43E-07 |
| SPIB_ T56 | 5.83E-07 |
| C5aR_ S338 | 1.76E-05 |
| ETV1_ S191 | 0.011974 |
