# Supplementary material for: Peptide microarray of pediatric acute myeloid leukemia is related to relapse and reveals involvement of DNA damage response and repair
Source: Oncotarget. 2019 Jul 23;10(45):4679–90. doi: 10.18632/oncotarget.27086 (PMC6659796; doi:10.18632/oncotarget.27086)
Supplement: Supplementary file 3 [file oncotarget-10-4679-s003.docx]

**Supplementary Table 3:** The 20 most significantly altered pathways of 192 activated peptides, upregulated peptides in cluster-1 and upregulated peptides in cluster-2.

| **# Serial** | | **Top 20 Pathways using upregulated peptides in cluster-1** | | | **p-value** | | **Network objects from active data** |
| --- | --- | --- | --- | --- | --- | --- | --- |
| 1 | | [Immune response _IFN gamma signaling pathway](http://portal.genego.com/cgi/imagemap.cgi?id=432) | | | 3.596E-09 | | p21, PKR, RAP-1A, p38 MAPK, AKT(PKB), SHP-2, STAT1, C/EBPbeta |
| 2 | | [DNA damage_Inhibition of telomerase activity and cellular senescence](http://portal.genego.com/cgi/imagemap.cgi?id=4862) | | | 3.840E-09 | | p21, AKT(PKB), p53, E2F1, p107, p130 |
| 3 | | [Effect of H. pylori infection on gastric epithelial cell proliferation](http://portal.genego.com/cgi/imagemap.cgi?id=3122) | | | 6.477E-09 | | c-Abl, p21, RAP-1A, p53, SHP-2, Cyclin D1, HGF receptor (Met), CREB1 |
| 4 | | [Neuroprotective action of lithium](http://portal.genego.com/cgi/imagemap.cgi?id=5634) | | | 1.272E-08 | | p38 MAPK, NR2B, HSF1, NR2A, p53, JNK1(MAPK8), CREB1, NR2 |
| 5 | | [Cell cycle_Regulation of G1/S transition (part 2)](http://portal.genego.com/cgi/imagemap.cgi?id=474) | | | 2.206E-08 | | Cyclin D, AKT(PKB), Cyclin D1, E2F1, p107, p130 |
| 6 | | [Development_G-CSF signaling](http://portal.genego.com/cgi/imagemap.cgi?id=6413) | | | 4.736E-08 | | JNK(MAPK8-10), p38 MAPK, AKT(PKB), SHP-2, p47-phox, STAT1, C/EBPbeta |
| 7 | | [IL-6 signaling in multiple myeloma](http://portal.genego.com/cgi/imagemap.cgi?id=4920) | | | 6.312E-08 | | p21, AKT(PKB), p53, SHP-2, STAT1, Cyclin D1, E2F1 |
| 8 | | [Neurophysiological process_NMDA-dependent postsynaptic long-term potentiation in CA1 hippocampal neurons](http://portal.genego.com/cgi/imagemap.cgi?id=3040) | | | 8.654E-08 | | RAP-1A, AKT(PKB), p90Rsk, NR2B, NR2A, mGluR1, CREB1, NR2 |
| 9 | | [Aberrant B-Raf signaling in melanoma progression](http://portal.genego.com/cgi/imagemap.cgi?id=6607) | | | 1.082E-07 | | p21, p90RSK1, MAP2, JNK(MAPK8-10), AKT(PKB), p90Rsk, AKT1 |
| 10 | | [Development_Non-genomic action of Retinoic acid in cell differentiation](http://portal.genego.com/cgi/imagemap.cgi?id=6895) | | | 1.229E-07 | | p90RSK1, AKT(PKB), p90Rsk, p38gamma (MAPK12), JNK1(MAPK8), CREB1, RhoA |
| 11 | | [Immune response_Oncostatin M signaling via MAPK in mouse cells](http://portal.genego.com/cgi/imagemap.cgi?id=2205) | | | 1.479E-07 | | PPAR-gamma, JNK(MAPK8-10), p38 MAPK, SHP-2, STAT1, C/EBPbeta |
| 12 | | [Development_Prolactin receptor signaling](http://portal.genego.com/cgi/imagemap.cgi?id=545) | | | 1.576E-07 | | AKT(PKB), SHP-2, VAV-2, STAT1, Cyclin D1, C/EBPbeta, RhoA |
| 13 | | [Immune response_Oncostatin M signaling via MAPK in human cells](http://portal.genego.com/cgi/imagemap.cgi?id=2204) | | | 2.094E-07 | | PPAR-gamma, JNK(MAPK8-10), p38 MAPK, SHP-2, STAT1, C/EBPbeta |
| 14 | | [Development_A2A receptor signaling](http://portal.genego.com/cgi/imagemap.cgi?id=643) | | | 5.310E-07 | | RAP-1A, JNK(MAPK8-10), p38 MAPK, AKT(PKB), p90Rsk, CREB1 |
| 15 | | [Mucin expression in CF airways](http://portal.genego.com/cgi/imagemap.cgi?id=2654) | | | 5.316E-07 | | p90RSK1, JNK(MAPK8-10), p38 MAPK, AKT(PKB), p90Rsk, JNK1(MAPK8), CREB1 |
| 16 | | [Signal transduction_PTMs in IL-17-induced CIKS-independent signaling pathways](http://portal.genego.com/cgi/imagemap.cgi?id=6603) | | | 8.023E-07 | | JNK(MAPK8-10), p38 MAPK, p90Rsk, AKT1, STAT1, C/EBPbeta |
| 17 | | [Development_Role of Thyroid hormone in regulation of oligodendrocyte differentiation](http://portal.genego.com/cgi/imagemap.cgi?id=4792) | | | 1.039E-06 | | p21, AKT(PKB), p53, AKT1, Cyclin D1, E2F1 |
| 18 | | [Immune response_C5a signaling](http://portal.genego.com/cgi/imagemap.cgi?id=6453) | | | 1.331E-06 | | JNK(MAPK8-10), p38 MAPK, AKT(PKB), p90Rsk, p47-phox, CREB1 |
| 19 | | [Apoptosis and survival_p53-dependent apoptosis](http://portal.genego.com/cgi/imagemap.cgi?id=428) | | | 1.682E-06 | | c-Abl, RAD9, JNK(MAPK8-10), p53, E2F1 |
| 20 | | [Cell cycle_Influence of Ras and Rho proteins on G1/S Transition](http://portal.genego.com/cgi/imagemap.cgi?id=4583) | | | 1.891E-06 | | p21, AKT(PKB), Cyclin D1, E2F1, JNK1(MAPK8), RhoA |
|  | |  | | |  | |  |
| **Serial** | | **Top 20 Pathways using upregulated peptides in cluster-2** | **p-value** | | **Network objects from active data** | |  |
| 1 | | [Development_IGF-1 receptor signaling](http://portal.genego.com/cgi/imagemap.cgi?id=540) | 5.367E-11 | | BAD, NF-kB, GYS1, Tuberin, c-Myc, c-Raf-1, FOXO3A, Elk-1, ASK1 (MAP3K5), RPS6 | |  |
| 2 | | [Immune response_C5a signaling](http://portal.genego.com/cgi/imagemap.cgi?id=6453) | 9.729E-10 | | BAD, PLD1, NF-kB, c-Raf-1, C5AR, G-protein alpha-i family, p47-phox, Rac1, PLC-beta | |  |
| 3 | | [Signal transduction_AKT signaling](http://portal.genego.com/cgi/imagemap.cgi?id=554) | 1.623E-07 | | BAD, NF-kB, GYS1, Tuberin, c-Myc, FOXO3A, RPS6 | |  |
| 4 | | [Development_Thrombopoietin-regulated cell processes](http://portal.genego.com/cgi/imagemap.cgi?id=631) | 2.250E-07 | | BAD, FKHR, c-Myc, c-Raf-1, Elk-1, Rac1, ATF-1 | |  |
| 5 | | [Immune response_Delta-type opioid receptor signaling in T-cells](http://portal.genego.com/cgi/imagemap.cgi?id=2665) | 2.912E-07 | | c-Src, Delta-type opioid receptor, c-Raf-1, Elk-1, G-protein alpha-i family, Rac1 | |  |
| 6 | | [Development_PIP3 signaling in cardiac myocytes](http://portal.genego.com/cgi/imagemap.cgi?id=701) | 3.070E-07 | | BAD, GYS1, Tuberin, c-Myc, FOXO3A, RPS6, 14-3-3 | |  |
| 7 | | [Development_A1 receptor signaling](http://portal.genego.com/cgi/imagemap.cgi?id=642) | 7.175E-07 | | c-Src, NF-kB, PLC-beta3, c-Raf-1, Elk-1, G-protein alpha-i family, Rac1 | |  |
| 8 | | [Immune response_Platelet activating factor/ PTAFR pathway signaling](http://portal.genego.com/cgi/imagemap.cgi?id=7056) | 9.298E-07 | | c-Src, NF-kB, NF-AT1(NFATC2), ASK1 (MAP3K5), G-protein alpha-i family, PLC-beta, NF-AT | |  |
| 9 | | [Development_Beta-adrenergic receptors transactivation of EGFR](http://portal.genego.com/cgi/imagemap.cgi?id=2433) | 1.339E-06 | | c-Src, Tuberin, c-Raf-1, G-protein alpha-i family, CD44, Beta-2 adrenergic receptor | |  |
| 10 | | [Development_c-Kit ligand signaling pathway during hemopoiesis](http://portal.genego.com/cgi/imagemap.cgi?id=6510) | 1.906E-06 | | BAD, PLD1, c-Raf-1, FOXO3A, Elk-1, GAB2, Rac1 | |  |
| 11 | | [Chemotaxis_C5a-induced chemotaxis](http://portal.genego.com/cgi/imagemap.cgi?id=6454) | 3.349E-06 | | PLD1, c-Raf-1, C5AR, G-protein alpha-i family, Rac1, PLC-beta | |  |
| 12 | | [Immune response_MIF-induced cell adhesion, migration and angiogenesis](http://portal.genego.com/cgi/imagemap.cgi?id=6564) | 5.026E-06 | | c-Src, NF-kB, c-Raf-1, G-protein alpha-i family, CD44, Rac1 | |  |
| 13 | | [Immune response_IL-2 activation and signaling pathway](http://portal.genego.com/cgi/imagemap.cgi?id=430) | 7.330E-06 | | NF-kB, c-Myc, c-Raf-1, NF-AT1(NFATC2), Elk-1, GAB2 | |  |
| 14 | | [Development_A3 receptor signaling](http://portal.genego.com/cgi/imagemap.cgi?id=644) | 7.330E-06 | | NF-kB, c-Myc, c-Raf-1, Elk-1, G-protein alpha-i family, PLC-beta | |  |
| 15 | | [Apoptosis and survival_nAChR in apoptosis inhibition and cell cycle progression](http://portal.genego.com/cgi/imagemap.cgi?id=3238) | 7.850E-06 | | c-Src, FKHR, Tuberin, c-Raf-1, Rb protein | |  |
| 16 | | [Development_EDNRB signaling](http://portal.genego.com/cgi/imagemap.cgi?id=2273) | 8.264E-06 | | c-Src, c-Raf-1, Elk-1, G-protein alpha-i family, ATF-1, PLC-beta | |  |
| 17 | | [PDE4 regulation of cyto/chemokine expression in inflammatory skin diseases](http://portal.genego.com/cgi/imagemap.cgi?id=6623) | 8.264E-06 | | NF-kB p50/p50, c-Raf-1, NF-AT1(NFATC2), G-protein alpha-i family, NF-kB1 (p50), 14-3-3 | |  |
| 18 | | [Signal transduction_PTMs in BAFF-induced signaling](http://portal.genego.com/cgi/imagemap.cgi?id=6664) | 9.293E-06 | | BAD, FKHR, Tuberin, c-Raf-1, FOXO3A, RPS6 | |  |
| 19 | | [DNA damage_Brca1 as a transcription regulator](http://portal.genego.com/cgi/imagemap.cgi?id=525) | 9.348E-06 | | Brca1, c-Myc, ATF-1, Rb protein, Chk2 | |  |
| 20 | | [Signal transduction_Activation of PKC via G-Protein coupled receptor](http://portal.genego.com/cgi/imagemap.cgi?id=453) | 1.042E-05 | | c-Src, NF-kB, c-Raf-1, NF-AT1(NFATC2), Elk-1, PLC-beta | |  |
