# Supplementary material for: Peptide microarray of pediatric acute myeloid leukemia is related to relapse and reveals involvement of DNA damage response and repair
Source: Oncotarget. 2019 Jul 23;10(45):4679–90. doi: 10.18632/oncotarget.27086 (PMC6659796; doi:10.18632/oncotarget.27086)
Supplement: Supplementary file 4 [file oncotarget-10-4679-s004.docx]

**Supplementary Table** **4:** List of 192 peptides that were highly activated in cluster-1 (C1) and cluster-2 (C2) with peptide name with p-site, sequence and with their functions. Peptides are sorted based on the functions and presented.

| **Name_P-site** | **Sequence** | **Clusters** | **Functions** |
| --- | --- | --- | --- |
| CCND1_ S90 | LDRFLSLEPVK | C1 | Cell cycle |
| E2F1_ S364 | LSRMGSLRAPV | C1 | Cell cycle |
| p21Cip1_ S146 | KRRQTSMTDFY | C1 | Cell cycle |
| RhoA_ S188 | RGKKKSGALVL | C1 | Cell cycle |
| CDC25B_ S146 | RLLGHSPVLRN | C2 | Cell cycle |
| CDC6_ S54 | KALPLSPRKRL | C2 | Cell cycle |
| CHK1_ S280 | RPRVTSGGVSE | C2 | Cell cycle |
| CHK2_ S19 | VLAQPSTSRKR | C2 | Cell cycle |
| RAC1_ S71 | RLRPLSYPQTV | C2 | Cell cycle |
| RAD9_ S336 | PQPPKSPGPHS | C2 | Cell cycle |
| B-Myb_ S577 | PGLRRSPIKKV | C2 | Cell cycle |
| BRCA1_ S509 | RKRRPTSGLHP | C2 | Cell cycle |
| CDC34_ S203 | APDEGSDLFYD | C1 | Cell cycle/Differentiation |
| RB1_ S807 | GNIYISPLKSP | C1 | Cell cycle/Proliferation |
| RB1_ S780 | RPPTLSPIPHI | C2 | Cell cycle/Proliferation |
| RB1_ S795 | YKFPSSPLRIP | C2 | Cell cycle/Proliferation |
| RBL2_ S1035 | DAPPLSPYPFV | C2 | Cell cycle/Proliferation |
| Rb-like 1_ S964 | DAPPLSPFPHI | C2 | Cell cycle/Proliferation |
| Rb-like 1_ S975 | KQQPGSPRRIS | C2 | Cell cycle/Proliferation |
| c-Myc_ S62 | RSGLASPSYVA | C2 | Cell cycle/Proliferation |
| c-Myc_ T58 | PTPPLSPSRRS | C2 | Cell cycle/Proliferation |
| CK1E_ S323 | GQLRGSATRAL | C2 | Cell cycle/WNT signaling |
| 5-LO_ S271 | LERQLSLEQEV | C1 | Proliferation |
| ADAM 12_ Y907 | PRSTHTAYIK | C1 | Proliferation |
| BMX_ Y40 | KTNLSYYEYDK | C1 | Proliferation |
| C/EBP-beta_ T235 | IAVRKSRDKAK | C1 | Proliferation |
| p47-phox_ S304 | PPRRSSIRNAH | C1 | Proliferation |
| PLCB3_ S1105 | RKRHNSISEAK | C1 | Proliferation |
| VRK1_ T305 | AKYMETVKLLD | C1 | Proliferation |
| VTN_ S381 | RKGYRSQRGHS | C1 | Proliferation |
| VTN_ S397 | NSRRPSRAMWL | C1 | Proliferation |
| 14-3-3-Eta_ S59;S60 | GARRSSWRVIS | C2 | Proliferation |
| DDX5_ S557 | AGIQTSFRTGN | C2 | Proliferation |
| FKHR_ T24 | RPRSATWPLPR | C2 | Proliferation |
| NF-κβ1_ S907 | HSLPLSPASTR | C2 | Proliferation |
| p40Phox_ T154 | RLRPRTRKVKS | C2 | Proliferation |
| p47-phox_ S320 | SRKRLSQDAYR | C2 | Proliferation |
| S6_ S236 | RRRLSSLRAST | C2 | Proliferation |
| Rap1A_ S180 | KKPKKKSALLL | C1 | Proliferation/Cell cycle |
| Rap1GAP1_ S484 | IVPGKSPTRKK | C1 | Proliferation/Cell cycle |
| c-Src_ S12 | KPKDASQRRRS | C2 | Proliferation/Cell cycle |
| Rap 1B_ S179 | KARKKSSAQLL | C2 | Proliferation/Cell cycle |
| Btk_ S180 | LKPGSSHRKTK | C1 | Proliferation/Differentiation |
| PTPRA_ S189 | GSHSNSFRLSN | C2 | Proliferation/Differentiation |
| STAT1_ S727 | NLLPMSPEEFD | C1 | Proliferation/Differentiation/Transcription factor |
| CD19_ Y409 | MRGILYAAPQL | C1 | Differentiation |
| CEACAM1_ Y493 | PTQPTSASPSL | C1 | Differentiation |
| CD44_ S291 | RDRHLSFSGSG | C2 | Differentiation |
| SHP2_ S591 | GLMQQQKSFR | C1 | Differentiation/Proliferation |
| SHP2_ Y542 | RKGHEYTNIKY | C1 | Differentiation/Proliferation |
| SMAD2_ T220 | NYIPETPPPGY | C2 | Differentiation/Proliferation/Apoptosis |
| BAD_ S75 | RSRHSSYPAGT | C2 | Apoptosis |
| Calpain-1_ T80 | TWRRGSTAGGA | C2 | Apoptosis |
| PAK6_ S560 | VPKRKSLVGTP | C2 | Apoptosis |
| p53_ S20 | SQETFSDLWKL | C1 | Apoptosis/Cell cycle |
| p53_ S9 | PQSDPSVEPPL | C1 | Apoptosis/Cell cycle |
| HSF1_ S230 | YSRQFSLEHVH | C1 | Apoptosis/Transcription factor |
| HSF1_ S303 | KEEPPSPPQSP | C1 | Apoptosis/Transcription factor |
| AKT1_ S473 | HFPQFSYSASS | C1 | PI3K/AKT |
| GAB2_ S159 | RERKSSAPSHS | C2 | PI3K/AKT |
| FOXO3A_ T32 | RPRSATWPLQR | C2 | PI3K/AKT and Cell cycle |
| eIF2-alpha_ S52 | LLSELSRRRIR | C2 | PI3K/AKT and Transcription |
| MAPK12_ Y185 | SEMTGYVVTRW | C1 | MAPK |
| ASK1_ S83 | RGRGSSVGGGS | C2 | MAPK |
| RAF1_ S259 | RQRSTSTPNVH | C2 | MAPK |
| ELK3_ S357 | FWSSLSPVAPL | C1 | MAPK and PI3K/AKT |
| ELK1_ S383 | FWSTLSPIAPR | C2 | MAPK and PI3K/AKT |
| ELK3_ S363 | PVAPLSPARLQ | C2 | MAPK and PI3K/AKT |
| HGFR_ Y1235 | YDKEYYSVHNK | C1 | MAPK and PI3K/AKT |
| ErbB3_ S1123 | RSRSRSPRPRG | C2 | MAPK and PI3K/AKT |
| JNK1_ Y223 | FMMTPYVVTRY | C1 | MAPK/apoptosis/Proliferation/Differentiation |
| p90RSK_ S227 | EKKAYSFAGTV | C1 | MAPK/Proliferation |
| APLP2_ T723 | KRQYGTISHGI | C1 | Metabolism |
| MOR-1_ S268 | SVRMLSGSKEK | C1 | Metabolism |
| PPP1R3A_ S48 | RRGSDSSEDIY | C1 | Metabolism |
| ACLY_ S450 | STPAPSRTASF | C2 | Metabolism |
| ATP2B1_ S1178 | TKRNSSPPPSP | C2 | Metabolism |
| GFAT_ S205 | GTRRGSPLLIG | C2 | Metabolism |
| GYS1_ S645 | ASVPPSPSLSR | C2 | Metabolism |
| PDE3B_ S295 | RPRRRSSAVSL | C2 | Metabolism |
| PDE3B_ S318 | IFRRPSLPAIS | C2 | Metabolism |
| PDE4B_ S133 | SQRRESFLYRS | C2 | Metabolism |
| PFKFB3_ S483 | RPRNYSVGSRP | C2 | Metabolism |
| PFKFB3_ T475 | LSSSNTIRRPR | C2 | Metabolism |
| PLD1_ S561 | KFSKFSLYKQL | C2 | Metabolism |
| PPP2R5A_ S28 | GFTRKSVRKAQ | C2 | Metabolism |
| TSC2_ S939 | RARSTSLNERP | C2 | Metabolism/Autophagy |
| LKB1_ S424 | KIRRLSAAKQQ | C2 | Metabolism/Autophagy |
| CREB_ S133 | LSRRPSYRKIL | C1 | Transcription factor |
| ATF-1_ S63 | LARRPSYRKIL | C2 | Transcription factor |
| EP300_ S89 | LLRSGSSPNLN | C2 | Transcription factor |
| SIIR_ S37 | SPPRSSLRRSS | C2 | Transcription factor |
| SPIB_ T56 | PPVPATPYEAF | C2 | Transcription factor |
| ADRB2_ S262 | GLRRSSKFALK | C1 | GPCR signaling |
| ADRBK1_ S670 | KNKPRSPVVEL | C1 | GPCR signaling |
| BKR2_ S373 | GTLRTSISVER | C1 | GPCR signaling |
| C5aR_ S314 | GRLRKSLPSLL | C1 | GPCR signaling |
| FPR2_ S236 | KGMIKSSRPLR | C1 | GPCR signaling |
| GluR4_ S862 | NKARLSITGSV | C1 | GPCR signaling |
| mGluR1_ T695 | KKKIATRKPRF | C1 | GPCR signaling |
| NMDAR2A_ Y1105 | EVERTYLKTKS | C1 | GPCR signaling |
| PKR_ T451 | TRSKGTLRYMS | C1 | GPCR signaling |
| RGS2_ S46 | WKTRLSYFLQN | C1 | GPCR signaling |
| RGS7_ S434 | LMKSDSYPRFI | C1 | GPCR signaling |
| 5-HT(2C)_ S456 | SVVSERISSV | C2 | GPCR signaling |
| ADRA2A_ S232 | TRVPPSRRGPD | C2 | GPCR signaling |
| ADRBK1_ S685 | PLVQRGSANGL | C2 | GPCR signaling |
| C5aR_ S338 | KSFTRSTVDTM | C2 | GPCR signaling |
| CCR5_ S336 | APERASSVYTR | C2 | GPCR signaling |
| PKN_ S374 | GLYSRSGSLSG | C2 | GPCR signaling |
| PKR_ S242 | RKAKRSLAPRF | C2 | GPCR signaling |
| RGS10_ S176 | AAKRASRIYNT | C2 | GPCR signaling |
| RYR2_ S2808 | RTRRISQTSQV | C2 | GPCR signaling |
| Dematin_ S403 | NELKKKASLF | C1 | Cytoskeletal |
| Fascin 1_ S38 | NASASSLKKKQ | C1 | Cytoskeletal |
| KIF1C_ S1092 | MRRQRSAPDLK | C1 | Cytoskeletal |
| KRT18_ S52 | VSRSTSFRGGM | C1 | Cytoskeletal |
| KRT8_ S431 | YGGLTSPGLSY | C1 | Cytoskeletal |
| KRT8_ S74 | NQSLLSPLVLE | C1 | Cytoskeletal |
| MAP2_ S1679 | KSKIGSTDNIK | C1 | Cytoskeletal |
| ADD3_ S693 | KFRTPSFLKKN | C2 | Cytoskeletal |
| SYN2_ S10 | LRRRLSDSSFI | C2 | Cytoskeletal |
| WIP_ S488 | ESRSGSNRRER | C2 | Cytoskeletal |
| ABL_ S465 466 | PGIDLSQVYEL | C1 | Oncogene |
| APC_ S2054 | KKKKPSRLKGD | C1 | WNT signaling |
| HLA-A_ S337 | WRRKSSDRKGG | C2 | Immune system |
| AQP0_ S331 | RLKSISERLSV | C1 | Other |
| CNPase_ S9 | GFSRKSHTFLP | C1 | Other |
| Coilin_ S184 | EAKRKSPKKKE | C1 | Other |
| CYTH1_ S394 | RKKKVSSTKRH | C1 | Other |
| Cytohesin 2_ S392 | RKKRISVKKKQ | C1 | Other |
| DARPP-32 _ T34 | RRRRPTPAMLF | C1 | Other |
| GNA15_ S336 | KKGARSRRLFS | C1 | Other |
| HCDH1_ S151 | SPYSLSPVSNK | C1 | Other |
| HMGA2_ S59 | GSKNKSPSKAA | C1 | Other |
| HMGCR_ S872 | MIHNRSKINLQ | C1 | Other |
| LEPR_ Y986 | QPFVKYATLIS | C1 | Other |
| LRP1_ Y4507 | FTNPVYATLYM | C1 | Other |
| MYF5_ S130 | IRYIESLQELL | C1 | Other |
| Neurogranin_ S36 | AKIQASFRGHM | C1 | Other |
| NR2B_ S1303 | LRRQHSYDTFV | C1 | Other |
| NUP210_ S1881 | PSGLWSPAYAS | C1 | Other |
| Occludin_ S340 | RFYPESSYKST | C1 | Other |
| PAM_ S930 | SRKGYSRKGFD | C1 | Other |
| PEA15_ S104 | LTRIPSAKKYK | C1 | Other |
| QDPR_ S223 | GKNRPSSGSLI | C1 | Other |
| RCAN1_ S112 | KQFLISPPASP | C1 | Other |
| SASH3_ S27 | LQRSSSFKDFA | C1 | Other |
| SF1_ S20 | KKRKRSRWNQD | C1 | Other |
| SH2D3A_ Y95 | ALVHSYMTGRR | C1 | Other |
| snRNP70_ S137 | IHMVYSKRSGK | C1 | Other |
| STXBP1_ S313 | KDFSSSKRMNT | C1 | Other |
| TCFL1_ S41 | GFTEESGDDEY | C1 | Other |
| TEK_ Y992 | RGQEVYVKKTM | C1 | Other |
| TESK1_ S220 | LAVVGSPYWMA | C1 | Other |
| THOP1_ S643 | GMDYRSAILRP | C1 | Other |
| TPH1_ S58 | SKRRNSEFEIF | C1 | Other |
| TRPV4_ Y253 | RRAKHYVELLV | C1 | Other |
| VAV2_ Y172 | GGDDIYEDIIK | C1 | Other |
| AQP2_ S256 | VRRRQSVELHS | C2 | Other |
| Ataxin-1_ S776 | RKRRWSAPESR | C2 | Other |
| CACNB2_ S478 | QHRSSSSAPHH | C2 | Other |
| CaR_ T888 | VAARATLRRSN | C2 | Other |
| CRMP-2_ T555 | IPRRTTQRIVA | C2 | Other |
| CTNNG_ S2849 | GSRRGSFDATG | C2 | Other |
| Cx32_ S233 | RSNPPSRKGSG | C2 | Other |
| Cx43_ S367 | PSSRASSRASS | C2 | Other |
| Desmin_ S12 | SSQRVSSYRRT | C2 | Other |
| ETV1_ S191 | FRRQLSEPANS | C2 | Other |
| G-alpha(Z)_ S27 | HLRSESQRQRR | C2 | Other |
| HMG14_ S7 | EPKRRSARLSA | C2 | Other |
| HMG17_ S25 | EPQRRSARLSA | C2 | Other |
| hnRNP K_ S302 | GGRGGSRARNL | C2 | Other |
| KEL_ S63 | VHRDLSRDRPL | C2 | Other |
| Lamin A/C_ S22 | SSTPLSPTRIT | C2 | Other |
| Lamin B1_ S395 | LSPSPSSRVTV | C2 | Other |
| LMNB1_ S23 | PTTPLSPTRLS | C2 | Other |
| NEUROD1_ S274 | FDGPLSPPLSI | C2 | Other |
| NFAT1_ S268 | LPPGASPQRSR | C2 | Other |
| NOLC1_ S623 | GEKRASSPFRR | C2 | Other |
| NPRA_ S538 | LSGRGSNYGSL | C2 | Other |
| NPR-B_ S526 | GSSYGSLMTAH | C2 | Other |
| Opioid receptor_ S344 | RPDPSSFSRPR | C2 | Other |
| PIGR_ S637 | NVDRVSIRSYR | C2 | Other |
| PPAR gamma_ S112 | KVEPASPPYYS | C2 | Other |
| Rab4_ S204 | LRQLRSPRRAQ | C2 | Other |
| Telethonin_ S157 | LRRSLSRSMSQ | C2 | Other |
| TFII-I_ S412 | PFRRPSTYGIP | C2 | Other |
| TFII-I_ S668 | TKALQSPKRPR | C2 | Other |
| TOP2A_ S1213 | AEVLPSPRGQR | C2 | Other |
| Vimentin_ S26 | ASRPSSSRSYV | C2 | Other |
| Vimentin_ S72 | AVRLRSSVPGV | C2 | Other |
